# Acute Hepatitis in Infections Caused by Dengue Virus in Southern Punjab, Pakistan

**DOI:** 10.7759/cureus.3788

**Published:** 2018-12-28

**Authors:** Rizwan Ishtiaq, Ali Imran, Hashim Raza, Qudsia Anwar, Daniyal Ishtiaq, Aftab Jamil, Qazi Masroor Ali, Raheel Khan

**Affiliations:** 1 Internal Medicine, St. Vincent Mercy Medical Center, Toledo, USA; 2 Internal Medicine, Quaid-E-Azam Medical College, Bahawalpur, PAK; 3 Internal Medicine, Bahawal Victoria Hospital, Bahawalpur, PAK; 4 Emergency Medicine, Bahawal Victoria Hospital, Bahawalpur, PAK; 5 Internal Medicine, Rawalpindi Medical College, Rawalpindi, PAK

**Keywords:** acute hepatitis, dengue fever, dengue hemorrhagic fever, aminotransferase

## Abstract

Background

Dengue is the most common vector-borne disease worldwide. It poses a significant health burden in tropical and subtropical countries. Common clinical presentations include retro-orbital pain, fever, headache, nausea, vomiting and aches and pains in the body. A severe form of dengue fever is known as dengue hemorrhagic fever (DHF) that includes signs of hemorrhage. Besides the typical signs and symptoms, atypical presentations of dengue include myositis, hepatitis and encephalitis. Hepatic involvement in dengue has varied presentations. This study aims to highlight the importance of acute hepatitis, an atypical presentation in dengue patients.

Methods

We conducted a descriptive, cross-sectional study in the Medical Unit-1 of Bahawal Victoria Hospital, Bahawalpur, a tertiary-care hospital serving the area of Southern Punjab, Pakistan. The relevant medical records of 63 patients admitted with dengue-associated hepatitis to the Medical Unit-1 of Bahawal Victoria Hospital, Bahawalpur, between January 1, 2015 and December 1, 2016, were reviewed. Informed consent was given. Information regarding demographic variables and disease course was collected and analyzed.

Results

This study included 55 men (87.3%) and eight (12.7%) women. Fifty (79.3%) patients were diagnosed with dengue fever (DF). Thirteen patients were managed on the lines of DHF. Out of the total 63 patients, only six were locals. The common clinical presentations in these patients included high fever, retro-orbital pain, severe headache, rash, dark-colored urine, bleeding problems and hepatomegaly. Higher levels of aspartate aminotransferase (AST) were noted in comparison to alanine transferase (ALT). Despite the complicated clinical course in some patients, all patients were managed successfully and discharged, except one.

Conclusion

The frequency of acute hepatitis in dengue patients is high, especially in young men. Early diagnosis and prompt treatment are necessary for better prognosis. Although no specific treatment guidelines are available, supportive treatment in a timely fashion can prevent complications. Transfusion with packed cell volume (PCV) and N-acetyl cysteine (NAC) has produced promising results.

## Introduction

Dengue is the most common arboviral disease globally with an estimated annual incidence of 100 million cases of dengue fever (DF), 250,000 of dengue hemorrhagic fever (DHF) and 25,000 fatalities [1]. Approximately 3.6 billion people living in tropical and subtropical countries have been affected by dengue illness [2]. The dengue virus belongs to the family of Flaviviridae and is usually spread by the bite of the mosquito, *Aedes aegypti* [3]. This disease is also known as “breakbone fever” because of its classic presentation of influenza-like illness with spiking fevers, fatigue, retro-orbital pain, myalgia and headaches. The incubation period is typically 3-14 days [4]. Laboratory findings include thrombocytopenia, leukopenia, hyponatremia and hemoconcentration [5]. Classic DF is a self-limiting disease, but if not managed promptly, it can be dangerous requiring hospitalization and may be fatal in most cases. DHF is a severe form of DF with signs of hemorrhage. If uncontrolled, it can progress to end-organ damage and hypotension, which is referred to as dengue shock syndrome (DSS).

Dengue illness can present with atypical presentations as well. Liver failure or hepatitis, encephalitis and myositis are a few underreported manifestations [6-8]. Hepatic involvement in dengue has a diverse clinical presentation, ranging from a mild elevation of liver enzymes to fulminant liver failure [9-10]. Management of hepatitis in dengue illness is still a matter of debate. No specific antiviral treatment is available. Intravenous hydration using packed cell transfusion to improve oxygen delivery to the liver cells, use of N-acetyl cysteine (NAC) and symptomatic management of complications are the common management strategies [11-12].

Recently, in countries like Pakistan, India and Sri Lanka, a more significant portion of the population has been infected by the dengue virus [13]. The incidence of DHF and DSS has been increasing in Pakistan [14]. Dengue illness unfolded as an epidemic all over Pakistan in 2011. Approximately 250,000 people suffered from dengue virus infection, and an estimated 200 people died. Despite appropriate measures and improvisation in clinical management, the mortality rate was high because of the atypical manifestations in patients. According to a survey conducted in Lahore, Pakistan, in 2011, out of the reported 95 mortalities, 41 (43.1%) patients had developed severe liver involvement along the course of the disease [15]. Primary dengue infection was associated with death in 21 patients, whereas fatalities in the remaining 74 patients were linked to dengue-associated complications, including hepatic involvement. In this study, we aim to highlight hepatic involvement in dengue infections. Although not frequent, monitoring and timely diagnosis of hepatic involvement can prevent unnecessary death of patients suffering from dengue infection.

## Materials and methods

This descriptive, cross-sectional study was conducted in the Medical Unit-1 of Bahawal Victoria Hospital, Bahawalpur, a tertiary-care hospital serving the area of Southern Punjab, Pakistan. We reviewed the relevant medical records of 63 patients admitted with dengue-associated hepatitis to the Medical Unit-1 of Bahawal Victoria Hospital, Bahawalpur, between January 1, 2015 and December 1, 2016. This study was approved by the Institutional Review Board of Quaid-e-Azam Medical College, Bahawalpur. Patients or their guardians provided informed consent, and patient information remained confidential. Information regarding variables such as age, sex, social class, locality of the patient, travel history, duration of onset of fever, presentation in the hospital, disease course and outcome were collected. Clinical symptoms and laboratory tests including liver function tests and serum electrolyte levels were performed regularly. Viral hepatitis testing was also performed to check for any co-infection or preexisting viral hepatitis. Supportive treatment in the form of intravenous and oral fluids and vitamin K was given to the patients. Statistical analysis was performed using Statistical Package for Social Sciences (SPSS) version 23.0 (IBM Corp., Armonk, NY).

## Results

**Figure 1:**
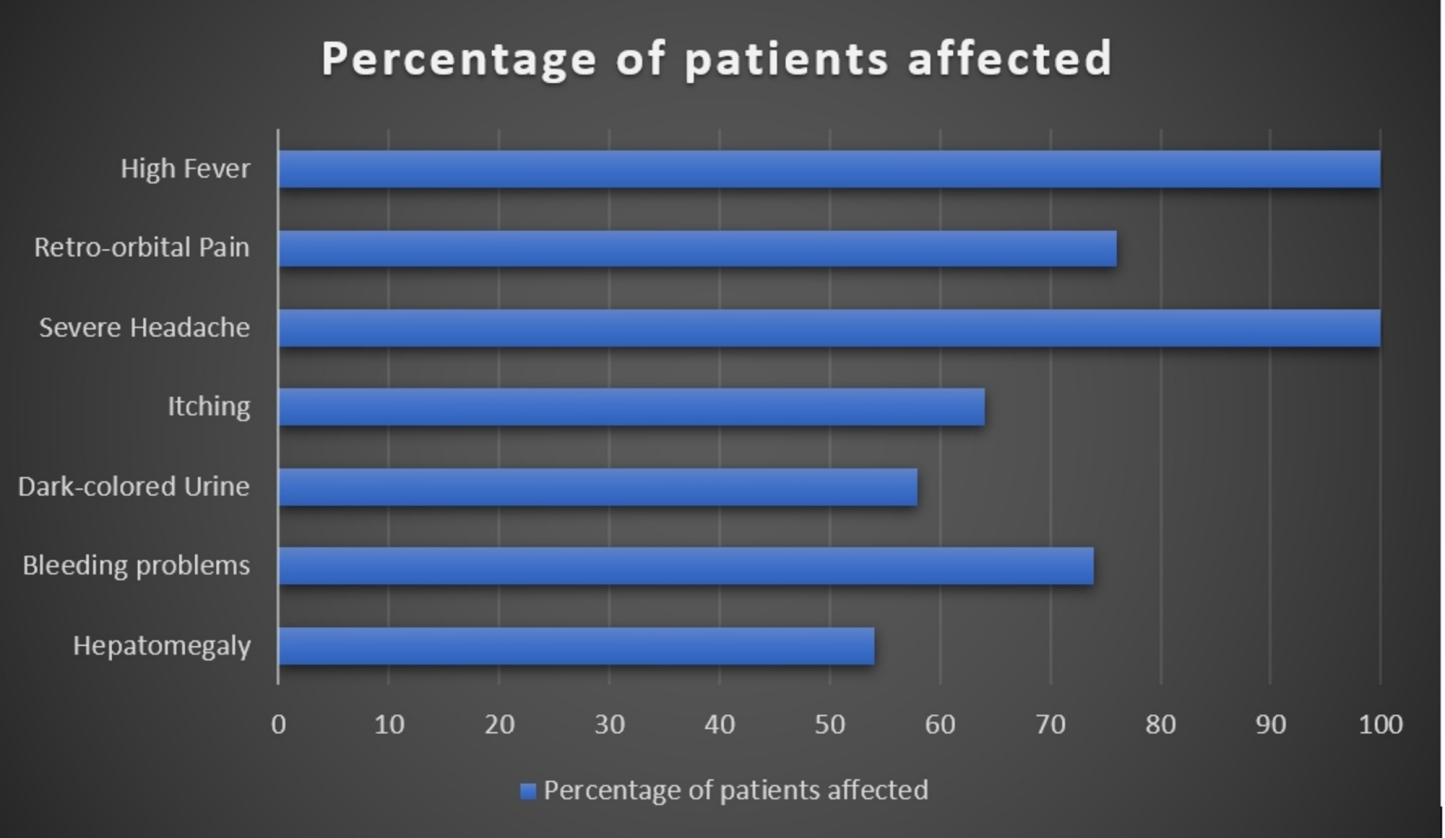
Common clinical presentations and their percentage frequencies in patients affected with dengue

This study included 55 men (87.3%) and eight (12.7%) women. The mean age of the patients was 29 ± 3.1 years. Most patients were young men of age 23-29 years. Thirty-seven (58.7%) patients belonged to the low socio-economic class and the remaining 26 (41.2%) belonged to the middle class. Fifty (79.3%) patients were diagnosed with DF. In the remaining 13 cases, five (7.9%) were diagnosed with DHF and eight were suspected to have DHF. All the 13 patients were managed on the lines of DHF. Regarding patient locality, six (9.5%) were locals and 54 (85.7%) patients were referred to us from locations outside Bahawalpur, taking into consideration that a significant proportion of patients in this subgroup belonged to areas of Southern Punjab and were working outside Southern Punjab for occupational purposes. Majority of these patients came from Karachi. The locality or travel history of three (4.7%) patients was unknown. Common clinical presentations and their frequency in our patients have been depicted in Figure 1.

Considering high-risk patients, one patient developed renal failure and was treated by two sessions of dialysis, while another developed ischemic heart disease. One patient developed DSS, and another patient was already a known case of obstructive uropathy at the time of diagnosis. The mean ± standard deviations of aspartate aminotransferase (AST) levels and alanine aminotransferase (ALT) levels were 2466 ± 547 U/L and 1382 ± 454 U/L, respectively. Viral hepatitis testing was also performed to check for any co-infection or preexisting viral hepatitis. We found that 14 out of 63 patients were carriers of hepatitis B or C at the time of diagnosis of DF. Non-structural protein 1 (NS1) test was positive in 46 (73%) patients, negative in 16 (25.3%) patients and unknown in one (1.5%) case. Based on Immunoglobulin M (IgM) serology, all cases were detected as early as within 3-4 days after admission, with most cases being diagnosed on the day or the next day of admission. All patients in this study were managed successfully and discharged except one who left against medical advice.

## Discussion

There are four distinct serotypes of dengue virus (DENV 1-4), which are known to cause dengue illness. More hepatic damage is seen in DENV 3 and DENV 4 [16]. Infection with one serotype confers ultimate protection against the same serotype, but infection with a different serotype may have worse clinical outcomes and predispose the patients to severe complications, e.g., plasma leak [17]. Symptoms like yellowness of skin and eyes, abdominal pain, itchy skin, nausea, vomiting, loss of appetite and dark-colored urine in patients with dengue infection suggest liver involvement [9]. In this study, patients mostly complained of hepatomegaly (54%), itchy skin (64%) and dark-colored urine (58%). Approximately 74% (*n* = 47) of patients tended to bruise easily. All these complaints are pathognomonic of liver injury. Hepatomegaly is more commonly present in DF, although it is present in DHF as well [18]. This finding is consistent with the current study. According to a study conducted on dengue patients in Vietnam, hepatic involvement is found in all patients with dengue and is in close correlation with disease severity [19]. According to Bowman et al., hepatic involvement in dengue is found more commonly in women, children or patients with a severe form of dengue [4]. Contrarily, we found hepatic involvement more commonly in male teenage patients without any critical form of DF.

Increased levels of aminotransferases can be seen in 90% of the patients with dengue with levels of AST higher than ALT [20]. As AST is also found in non-hepatic sites like red blood cells and muscles, this trend of higher AST can be attributed to the involvement of muscle breakdown and hemolysis in DF. Supporting this observation, a study evaluated approximately 270 patients with dengue [21]. According to this study, nearly 90% of the patients had abnormal levels of AST in comparison to ALT, gamma-glutamyl transferase, alkaline phosphatase and bilirubin (80%, 83%, 16% and 7%, respectively). Another study conducted in 1,585 patients in Brazil confirmed greater alterations in the AST levels compared to ALT (63.4% vs. 45%) [10]. The present study also showed increased levels of AST compared to ALT.

It is difficult to diagnose the co-infection of dengue virus with hepatitis-causing viruses and manage patients due to the accelerated compromise in liver functions. Three cases of co-infection of dengue virus with hepatitis A have been reported in the literature [22]. It is necessary to rule out both conditions when a patient affected with dengue presents with acute hepatitis because the clinical pictures are superimposed on each other. Coagulation profile and serum aminotransferase levels can help differentiate acute hepatitis caused by dengue or viral hepatitis. Dengue causing hepatitis does not usually affect the coagulation profile in contrast to viral hepatitis [23]. In dengue, serum aminotransferase levels are usually found to be 2-3 times the normal value in comparison to viral hepatitis with values 8-10 times the normal level [24]. Other factors that can help in differentiation include thrombocytopenia and hemoconcentration [25]. A study in China calculated the difference in the levels of interleukin in patients with co-infections of dengue and hepatitis B [26]. Patients with co-infections were found to have less amounts of interleukin (IL)-6 and tumor necrosis factor. In the present study, none of the patients suffered from newly diagnosed viral hepatitis during their disease course. However, 14 patients had ongoing hepatitis B or C infections.

There are conflicting guidelines for the treatment of hepatitis caused by dengue virus as there is no available antidote. Transfusion by packed cells is recommended to increase the PCV [27]. It also helps increase the oxygen-carrying capacity and recover the liver cells from necrosis. NAC infusion removes free radicals, improves antioxidant effects and acts as a vasodilator improving oxygen supply [28]. Data regarding the efficacy of NAC in dengue-associated liver damage are limited. Large clinical studies need to be done in this regard. Intake of acetaminophen for fever in dengue infection has also been suggested to accelerate liver damage [29]. In the present study, 39 patients were already taking acetaminophen. One of them reported taking four pills of acetaminophen for six days before developing a liver injury.

Due to the recent outbreaks of dengue, the need for advanced and efficient vaccines has grown more urgent to control such outbreaks. Several advancements have already been made in vaccines for the adolescent and adult population. A phase II clinical trial sponsored by the Butantan Institute, Brazil, is currently active to evaluate the safety and immunogenicity of a tetravalent lyophilized dengue vaccine. Another clinical trial sponsored by the National Institute of Allergy and Infectious Diseases (NIAID) is currently recruiting participants to evaluate the ability of a single dose of live accentuated vaccine (TetraVax-DV-TV005) to protect against infection with serotype DENV-3 of DF. Under the sponsorship of the U.S. Army Medical Research and Materiel Command, another clinical trial is currently underway to study the safety of a vaccine (DENV-1 PIV) for the prevention of DF. Such vaccines will have significant health and economic impact across the globe.

The limitations of this study include that data was obtained from a single source, i.e., Bahawal Victoria Hospital, which can be a source of bias in sample selection. This limitation can be overcome by conducting multicenter studies comparing fatal vs. nonfatal cases and clinical and laboratory findings.

## Conclusions

This study aimed to highlight the importance of hepatitis in patients with dengue infection. It is also important to review the history of medication intake in patients with dengue fever (DF) who present with hepatitis as drugs like acetaminophen used for fever can also cause hepatic damage. If a patient is undertaking such medication, then its intake should be regularly checked. Management with transfusing packed cell volume (PCV) of blood is essential to improve oxygen supply to the virally damaged liver cells as it helps treat the necrotic liver cells without developing into fulminant liver failure. Awareness regarding primary prevention of dengue virus should be spread with the help of electronic and social media. Vector control activities should be implemented at the state level.
